# First case report of vancomycin-intermediate sequence type 72 *Staphylococcus aureus*with nonsusceptibility to daptomycin

**DOI:** 10.1186/1471-2334-14-459

**Published:** 2014-08-23

**Authors:** Ayaka Tsukimori, Itaru Nakamura, Sakiko Okamura, Akihiro Sato, Shinji Fukushima, Yasutaka Mizuno, Tetsuo Yamaguchi, Tetsuya Matsumoto

**Affiliations:** Department of Infection Control and Prevention, Tokyo Medical University Hospital, 6-7-1 Nishishinjuku, Shinjuku-ku, Tokyo, 160-0023 Japan; Department of Microbiology, Tokyo Medical University, 6-1-1 Nishishinjuku, Shinjuku-ku, Tokyo, 160-8402 Japan; Department of Medical Treatment for Health Scientific Research, Bunkyo Gakuin University Graduate School, 1-19-1 Mukougaoka, Bunkyo-ku, Tokyo 113-8668 Japan; Department of Microbiology and Infectious Diseases, Toho University Faculty of Medicine, 5-21-16 Omorinishi, Oota-ku, Tokyo, 143-8540 Japan

**Keywords:** MRSA, Daptomycin, Sequence type 72-MRSA-SCC*mec* type IV (ST72-MRSA-IV), Daptomycin nonsusceptibility, Vancomycin-intermediate resistance, Rifampicin resistance, High-dose daptomycin

## Abstract

**Background:**

Sequence type 72 methicillin-resistant *Staphylococcus aureus* (MRSA) SCC*mec* type IV (ST72-MRSA-IV) is the most common community-acquired MRSA clone in Korea. Resistance to daptomycin or vancomycin among community-acquired MRSA clones is not well described in the literature. We herein report the first case of vancomycin-intermediate, daptomycin-nonsusceptible ST72-MRSA-IV.

**Case presentation:**

A 45-year-old Japanese man underwent aortic arch prosthesis implantation for treatment of a dissecting aortic aneurysm. Fourteen months later, he developed a prosthetic graft infection of the aortic arch and an anterior mediastinal abscess caused by ST72-MRSA-IV. First-line treatment with vancomycin and rifampicin failed, and daptomycin was thus administered. After several days, the treatment was changed to linezolid because of the re-emergence of fever. The patient’s condition resolved and no recurrence or other problems were seen for 1 year post-treatment. The infectious agent was definitively identified as vancomycin-intermediate, daptomycin-nonsusceptible, rifampicin-resistant ST72-MRSA-IV based on culture results and minimum inhibitory concentration testing.

**Conclusion:**

This case report illustrates the importance of fully understanding the changing epidemiology of infectious agents and the risk factors for the development of antibiotic resistance. Such information will help to minimize the emergence and spread of antibiotic-resistant strains. This report concerns one particular bacterial strain; however, the basic concepts involved in this case translate to all infectious disease fields.

**Electronic supplementary material:**

The online version of this article (doi:10.1186/1471-2334-14-459) contains supplementary material, which is available to authorized users.

## Background

Community-acquired methicillin-resistant *Staphylococcus aureus* (CA-MRSA) was first reported in 1981 in patients with MRSA infections without general healthcare-associated risk factors. The incidence of CA-MRSA infection has continually increased since the mid-1990s [1]. The features of CA-MRSA strains differ from those of healthcare-associated MRSA (HA-MRSA) strains. Reported differences between infections with these two strains include genetic traits, clinical features, patient groups, infection routes, recommended treatments and management, prevention procedures, and antimicrobial susceptibility patterns [2]. In recent years, however, these strains have been frequently observed in healthcare settings and have been shown to cause both community-onset healthcare-associated infections and nosocomial infections [1, 3].

CA-MRSA clones vary among geographic settings. Sequence type 8 (ST8)-staphylococcal cassette chromosome *mec* (SCC*mec*) type IV (ST8-MRSA-IV, known as USA300) is the most common and epidemic CA-MRSA clone in North America [4]. The most predominant CA-MRSA clones in Asian countries include ST59-MRSA-IV in Taiwan, Hong Kong, Vietnam, and Sri Lanka; ST30-MRSA-IV in the Philippines; and ST72-MRSA-IV in Korea. Clinical strains of ST72-MRSA-IV are mostly limited to Korea and lack Panton–Valentine leukocidin (PVL) genes. ST72-MRSA-IV isolates usually have very low resistance to most non-β-lactam agents, which is a typical characteristic of CA-MRSA isolates containing SCC*mec* type IV elements [5]. Multidrug resistance in ST72-MRSA-IV isolates has rarely been reported [6, 7]. ST72-MRSA-IV strains, like other CA-MRSA strains, commonly cause skin and soft tissue infections or pneumonia and have sometimes been associated with serious or fatal diseases such as severe sepsis, septic shock, and surgical site infection [8].

Daptomycin was first approved in 2003 for the treatment of complicated Gram-positive skin and skin structure infections. It is a novel lipopeptide antibiotic with a unique mechanism of action against Gram-positive bacteria, binding to bacterial membranes and causing rapid depolarization of the membrane potential [9]. Because daptomycin was confirmed to be effective against strains that were resistant to existing antibiotics, its clinical application has been increasing as the first-line therapy for bacteremia, surgical site infection, and right-sided infective endocarditis [10]. With the exception of USA300, resistance to daptomycin or vancomycin among CA-MRSA clones has not been well described [11, 12]. The first case of an ST72-MRSA-IV strain in Asia demonstrating vancomycin-intermediate resistance was reported in 2012 by Chung et al. in Korea [13]. To our knowledge, the present case is the first report of an ST72-MRSA-IV strain with daptomycin nonsusceptibility and vancomycin-intermediate resistance.

## Case presentation

### Clinical presentation

A 45-year-old Japanese man presented to the emergency department with the main complaints of fever and disturbance of consciousness (Glasgow Coma Score of E3V4M6). He had a previous medical history of dissecting aortic aneurysm (Stanford type A), for which he underwent transverse aortic arch replacement 14 months before admission. He had no history of travelling abroad or having his skin pierced. He had previously been treated with vancomycin at 3 g/day (1 g intravenously [IV] three times a day [TID]) and rifampicin at 600 mg/day (300 mg orally [PO] twice a day [BID]) for 2 days before being transferred to our hospital.

**Figure 1:**
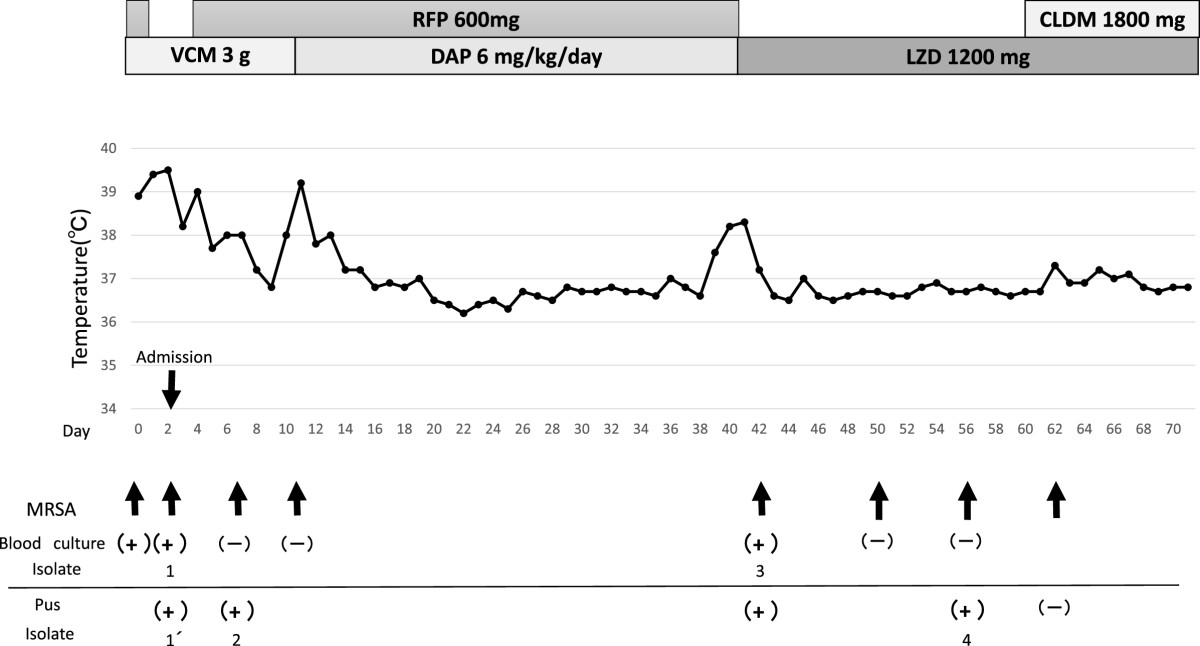
**Time course of body temperature variation and antibiotic regimen of the patient.** The patient’s blood culture became negative for MRSA once in the course of vancomycin therapy, but his fever persisted. With the initiation of daptomycin therapy, his temperature remained relatively low for several days and then increased when the blood culture became positive again for MRSA. 1: blood culture on the first hospital day; 1’: pus from the abscess on the first hospital day; 2: pus from the abscess on the 6th hospital day; 3: blood culture on the 40th hospital day; 4: pus from the abscess on the 55th hospital day. CEZ: cefazolin; VCM: vancomycin; RFP: rifampicin; DAP: daptomycin; LZD: linezolid; CLDM: clindamycin

The patient’s condition was diagnosed as an anterior mediastinal abscess and hemorrhagic cerebral infarction associated with a prosthetic graft infection of the aortic arch. Surgical intervention to remove the infected graft, which required anticoagulant therapy during cardiopulmonary bypass, was not possible because of intracerebral hemorrhage. Initial treatment for the prosthetic graft infection was started with vancomycin at 3 g/day (1 g IV TID) and cefazolin at 6 g/day (2 g IV TID). On the second hospital day, drainage and continuous irrigation were started for treatment of the anterior mediastinal abscess. Because the results of the blood culture performed 2 days before admission revealed an MRSA infection, the treatment was switched to vancomycin at 3 g/day (1 g IV TID) and rifampicin at 600 mg/day (300 mg PO BID); this treatment was continued for 9 days. The isolates from the blood and anterior mediastinal cultures performed on the day of admission also indicated MRSA infection. The vancomycin serum trough levels remained at 9.8–12.2 μg/ml during vancomycin treatment. The vancomycin minimum inhibitory concentration (MIC) of the MRSA strain isolated from the pus in the abscess was 2 μg/ml, and this strain was also susceptible to most non-β-lactam agents except gentamicin (Table 1). The blood culture became negative for MRSA during the course of vancomycin and rifampicin therapy. However, the patient became feverish again on the 10th hospital day, and the treatment was switched to IV daptomycin at 6 mg/kg/day and rifampicin. On the 40th day, after the patient had remained in a stable condition for several days with daptomycin and rifampicin treatment, his fever returned and the blood culture became positive again for MRSA. Therefore, we suspected nonsusceptibility to daptomycin. Daptomycin was discontinued and treatment with linezolid was initiated at 1200 mg/day (600 mg IV BID). Reconstruction of the acute aortic dissection was carried out on the 61st day because the chest computed tomography scan performed during therapy showed persistence of the anterior mediastinal abscess and gallium scintigraphy showed persistence of prosthetic graft-associated inflammation. The infected prosthetic graft was completely removed and replaced. After the surgery, antimicrobial treatment was switched to linezolid at 1200 mg/day (600 mg PO BID) and clindamycin at 1800 mg/day (600 mg PO TID). The patient’s condition subsequently improved, and recurrence was not observed for 12 months of follow-up. The time course of his body temperature variations and antibiotic treatment regimen are shown in Figure 1.

**Table 1:** **Susceptibility of MRSA to various antimicrobial agents analyzed by clinical laboratory testing**

Antimicrobial agent	MIC (μg/ml) [interpretation]				
	Isolate 1	Isolate 1’	Isolate 2	Isolate 3	Isolate 4
Oxacillin	>4 [R]	>4 [R]	>4 [R]	>4 [R]	>4 [R]
Gentamicin	8 [R]	>8 [R]	8 [R]	>8 [R]	>8 [R]
Erythromycin	<0.25 [S]	<0.25 [S]	<0.25 [S]	<0.25 [S]	<0.25 [S]
Clindamycin	<0.5 [S]	<0.5 [S]	<0.5 [S]	<0.5 [S]	<0.5 [S]
Minocycline	<2 [S]	<2 [S]	<2 [S]	<2 [S]	<2 [S]
Teicoplanin	<2 [S]	<2 [S]	<2 [S]	4 [S]	4 [S]
Vancomycin	1 [S]	2 [S]	1 [S]	2 [S]	4 [I]
Levofloxacin	<0.5 [S]	<0.5 [S]	<0.5 [S]	<0.5 [S]	<0.5 [S]
Rifampicin	<1 [S]	<1 [S]	>2 [R]	>2 [R]	>2 [R]

Susceptibilities to the agents in Table 1 were evaluated by broth microdilution (MicroScan; Siemens, Tokyo, Japan).

Isolate 1: blood culture on the first hospital day; Isolate1’: pus from the abscess on the first hospital day; Isolate 2: pus from the abscess on the sixth hospital day; Isolate 3: blood culture on the 40th hospital day; Isolate 4: pus from the abscess on the 55th hospital day. S: Susceptible; I: Intermediate; R: Resistant.

### Methods and results

Antimicrobial susceptibility testing was performed according to the Clinical and Laboratory Standards Institute (CLSI) guidelines (Tables 1 and 2). Rifampicin susceptibility testing, not routinely included, was performed after the treatment and revealed resistance at an MIC of >2 μg/ml from Isolate 2 in the course of vancomycin and rifampicin dosing (Table 1). The MRSA isolate from the pus in the abscess on the 55th hospital day (Isolate 4) demonstrated a vancomycin MIC of 4 μg/ml, indicating vancomycin-intermediate *S. aureus* (VISA) (Table 1). The daptomycin MICs of the MRSA isolates obtained from the blood and anterior mediastinal abscess cultures were evaluated by the broth microdilution method, using MicroScan (Siemens, Tokyo, Japan) and frozen plate (Eiken Chemical Co., Ltd., Tokyo, Japan), and Etest® (bioMérieux, Marcy-l'Étoile, France). The MRSA isolates showed susceptibility to daptomycin at the time of admission, with MICs of ≤0.5 μg/ml, 0.5, and 0.125 μg/ml upon analysis by MicroScan, frozen plate, and Etest®, respectively. Over time, however, these MICs increased to >1, 1.5, and 1.5 μg/ml, respectively, indicating daptomycin nonsusceptibility (Table 2). The interpretations of the MIC results (susceptible, intermediate, nonsusceptible, or resistant) were determined according to the CLSI guidelines. MRSA isolates with daptomycin MICs of >1 μg/ml, vancomycin MICs of 4 to 8 μg/ml, and rifampicin MICs of >2 μg/ml were defined as daptomycin nonsusceptible, vancomycin-intermediate resistant, and rifampicin resistant, respectively.

**Table 2:** **Susceptibility of MRSA to daptomycin as analyzed by clinical laboratory testing**

Method	Daptomycin MIC (μg/ml) [interpretation]				
	Isolate 1	Isolate 1’	Isolate 2	Isolate 3	Isolate 4
MicroScan (Siemens, Tokyo, Japan)	≤0.5 [S]	≤0.5 [S]	≤0.5 [S]	>1 [NS]	>1 [NS]
Frozen plate (Eiken, Tokyo, Japan)	0.5 [S]	0.5 [S]	0.25 [S]	1 [S]	1.5 [NS]
Etest® (bioMérieux, Marcy-l'Étoile, France)	0.125 [S]	0.125 [S]	0.125 [S]	1 [S]	1.5 [NS]

Isolate 1: blood culture on the first hospital day; Isolate1’: pus from the abscess on the first hospital day; Isolate 2: pus from the abscess on the sixth hospital day; Isolate 3: blood culture on the 40th hospital day; Isolate 4: pus from the abscess on the 55th hospital day. S: Susceptible; NS: Nonsusceptible.

Pulsed-field gel electrophoresis was performed using a contour-clamped homogeneous electric field dynamic regulation III system (CHEF-DR system; Bio-Rad Laboratories, Hercules, CA, USA) as previously described by McDougal et al. [14]. The results indicated that the isolates obtained sequentially in this case were derived from the same origin. Multilocus sequence typing (MLST) analysis was performed by sequencing seven housekeeping genes (*arcC*, *aroE*, *glpF*, *gmk*, *pta*, *tpi*, and *yqiL*) based on the MLST database (http://saureus.mlst.net), as described by Enright et al. [15]. The characterization of SCC*mec*[16] and the detection of PVL genes [17] were performed by polymerase chain reaction as described previously. Typing of the MRSA isolates revealed that they belonged to ST72 (1-4-1-8-4-4-3), carried SCC*mec* type IV, and were negative for PVL.

## Discussion

The antimicrobial susceptibility rates of 25 different ST72 isolates in Korea were previously reported as follows: erythromycin (4%), clindamycin (4%), ciprofloxacin (60%), TMP/SXT (96%), gentamicin (72%), rifampicin (92%), and vancomycin (100%) [6]. Although the ST72-MRSA-IV isolates in the present case were susceptible to most non-β-lactam agents (Table 1), they developed reduced susceptibility to three key drugs, namely vancomycin, daptomycin, and rifampicin.

The possible risk factors for the development of daptomycin nonsusceptible strains in our case, as in common MRSA strains, included the following: the presence of an intractable infectious disease (prosthetic graft infection of the aortic arch), a delay in the performance of necessary surgical care [18], previous exposure to vancomycin or a vancomycin MIC of ≥2 μg/ml [19], treatment with insufficient doses of daptomycin [20], and prolonged use of daptomycin [21]. Additionally, some reports have stated that VISA exhibits reduced susceptibility to daptomycin [22, 23].

Another possible factor was the presence of resistance to rifampicin, which was added to allow for vancomycin penetration into the biofilm [24]. The combined use of vancomycin and rifampicin is reportedly a potential risk factor in the treatment of MRSA [25]. Cui et al. confirmed that *rpoB* (RNA polymerase subunit β) mutation, known to be associated with rifampicin resistance [26], conferred dual heteroresistance to daptomycin and vancomycin in *S. aureus* and exhibited a thickened cell wall and reduction of the negative charge on the cell surface [27]. The mechanism of resistance to daptomycin remains unclear. However, considering that the present case involved a daptomycin-nonsusceptible VISA strain with rifampicin resistance, it is quite possible that the *rpoB* mutation, led by the combined treatment of vancomycin and rifampicin, may have contributed to the development of resistance.

Among the above-mentioned risk factors, our case involved those that could have been avoided or decreased by alternative therapy. An alternative approach to decreasing these risk factors could have been the administration of high-dose daptomycin upon the diagnosis of an intractable MRSA infection. Daptomycin exhibits rapid and concentration-dependent bactericidal activity, which translates into an increased rate of killing against most drug-susceptible strains and improved activity even against some nonsusceptible strains [20, 28, 29]. Rose et al. stated that high-dose daptomycin at 10 mg/kg/day was likely to prevent the emergence of increased daptomycin MICs [20, 29]. The safety and efficacy of daptomycin at 8 to 12 mg/kg/day for the treatment of complicated bacteremia have also been reported [28]. Additionally, early use of daptomycin at higher doses was proven to be effective when the initial vancomycin MIC showed susceptibility at >1 μg/ml [30]. Although the Infectious Diseases Society of America recommends an alternative to vancomycin for isolates with a vancomycin MIC of ≥2 μg/ml, we recommend an early treatment switch in patients with intractable disease with a vancomycin susceptibility level of >1 μg/ml.

## Conclusions

We have herein reported the first case of daptomycin nonsusceptibility among clinical ST72-MRSA-IV strains, which are rarely observed in countries other than Korea. In this case, failure to implement the appropriate dose and combination of antibacterial agents might have contributed to the development of nonsusceptibility. Further efforts to understand the changing epidemiology and the risk factors for antibiotic resistance will be necessary to minimize the future emergence or spread of antibiotic-resistant strains.

### Consent

Written informed consent was obtained from the patient for the publication of this case report and any accompanying images. A copy of the written informed consent is available for review by the Editor of this journal.

### Ethics statement

This study was performed in accordance with the Helsinki Declaration. No human experimentation was performed. No ethical approval from the ethics committee of Tokyo Medical University was required for this study.

## Authors’ original submitted files for images

Below are the links to the authors’ original submitted files for images. Authors’ original file for figure 1 Authors’ original file for figure 2

## Abbreviations

MRSA: Methicillin-resistant *Staphylococcus aureus*
CA: Community-acquired
SCC*mec*: Staphylococcal cassette chromosome *mec*
ST72-MRSA-IV: Sequence type 72 MRSA SCC*mec* type IV
HA: Healthcare-associated
VISA: Vancomycin-intermediate *Staphylococcus aureus*
MIC: Minimum inhibitory concentration
CLSI: Clinical and Laboratory Standards Institute
MLST: Multilocus sequence typing
PVL: Panton-Valentine leukocidin.

## References

[1] David MZ, Daum RS (2010). Community-associated methicillin-resistant *Staphylococcus aureus*: epidemiology and clinical consequences of an emerging epidemic. Clin Microbiol Rev.

[2] Naimi TS, LeDell KH, Como-Sabetti K, Borchardt SM, Boxrud DJ, Etienne J, Johnson SK, Vandenesch F, Fridkin S, O'Boyle C, Danila RN, Lynfield R (2003). Comparison of community- and health care-associated methicillin-resistant *Staphylococcus aureus* infection. JAMA.

[3] Nakamura I, Yamaguchi T, Miura Y, Shimizu H, Fukushima S, Mizuno Y, Matsumoto T (2013). Clinical aspects of infection with methicillin-resistant *Staphylococcus aureus* USA300 strain, generally regarded as community-acquired, in Japan. Jpn J Infect Dis.

[4] Tenover FC, Goering RV (2009). Methicillin-resistant *Staphylococcus aureus* strain USA300: origin and epidemiology. J Antimicrob Chemother.

[5] Song JH, Hsueh PR, Chung DR, Ko KS, Kang CI, Peck KR, Yeom JS, Kim SW, Chang HH, Kim YS, Jung SI, Son JS, So TM, Lalitha MK, Yang Y, Huang SG, Wang H, Lu Q, Carlos CC, Perera JA, Chiu CH, Liu JW, Chongthaleong A, Thamlikitkul V, Van PH (2011). Spread of methicillin-resistant *Staphylococcus aureus* between the community and the hospitals in Asian countries: an ANSORP study. J Antimicrob Chemother.

[6] Kim ES, Song JS, Lee HJ, Choe PG, Park KH, Cho JH, Park WB, Kim SH, Bang JH, Kim DM, Park KU, Shin S, Lee MS, Choi HJ, Kim NJ, Kim EC, Oh MD, Kim HB, Choe KW (2007). A survey of community-associated methicillin-resistant *Staphylococcus aureus* in Korea. J Antimicrob Chemother.

[7] Ko KS, Lim SK, Jung SC, Yoon JM, Choi JY, Song JH (2011). Sequence type 72 meticillin-resistant *Staphylococcus aureus* isolates from humans, raw meat and soil in South Korea. J Med Microbiol.

[8] Joo EJ, Chung DR, Ha YE, Park SY, Kim HA, Lim MH, Kim SH, Kang CI, Lee NY, Ko KS, Peck KR, Song JH (2012). Clinical predictors of community-genotype ST72-methicillin-resistant *Staphylococcus aureus*-SCC*mec* type IV in patients with community-onset *S. aureus* infection. J Antimicrob Chemother.

[9] Steenbergen JN, Alder J, Thorne GM, Tally FP (2005). Daptomycin: a lipopeptide antibiotic for the treatment of serious Gram-positive infections. J Antimicrob Chemother.

[10] Critchley IA, Draghi DC, Sahm DF, Thornsberry C, Jones ME, Karlowsky JA (2003). Activity of daptomycin against susceptible and multidrug-resistant Gram-positive pathogens collected in the SECURE study (Europe) during 2000–2001. J Antimicrob Chemother.

[11] Murthy MH, Olson ME, Wickert RW, Fey PD, Jalali Z (2008). Daptomycin non-susceptible meticillin-resistant *Staphylococcus aureus* USA 300 isolate. J Med Microbiol.

[12] Graber CJ, Wong MK, Carleton HA, Perdreau-Remington F, Haller BL, Chambers HF (2007). Intermediate vancomycin susceptibility in a community-associated MRSA clone. Emerg Infect Dis.

[13] Chung DR, Baek JY, Kim HA, Lim MH, Kim SH, Ko KS, Kang CI, Peck KR, Lee NY, Song JH (2012). First report of vancomycin-intermediate resistance in sequence type 72 community genotype methicillin-resistant *Staphylococcus aureus*. J Clin Microbiol.

[14] McDougal LK, Steward CD, Killgore GE, Chaitram JM, McAllister SK, Tenover FC (2003). Pulsed-field gel electrophoresis typing of oxacillin-resistant *Staphylococcus aureus* isolates from the United States: establishing a national database. J Clin Microbiol.

[15] Enright MC, Day NP, Davies CE, Peacock SJ, Spratt BG (2000). Multilocus sequence typing for characterization of methicillin-resistant and methicillin-susceptible clones of *Staphylococcus aureus*. J Clin Microbiol.

[16] Kondo Y, Ito T, Ma XX, Watanabe S, Kreiswirth BN, Etienne J, Hiramatsu K (2007). Combination of multiplex PCRs for staphylococcal cassette chromosome *mec* type assignment: rapid identification system for *mec,* ccr, and major differences in junkyard regions. Antimicrob Agents Chemother.

[17] Lina G, Piemont Y, Godail-Gamot F, Bes M, Peter MO, Gauduchon V, Vandenesch F, Etienne J (1999). Involvement of Panton-Valentine leukocidin-producing *Staphylococcus aureus* in primary skin infections and pneumonia. Clin Infect Dis.

[18] Fowler VG, Boucher HW, Corey GR, Abrutyn E, Karchmer AW, Rupp ME, Levine DP, Chambers HF, Tally FP, Vigliani GA, Cabell CH, Link AS, DeMeyer I, Filler SG, Zervos M, Cook P, Parsonnet J, Bernstein JM, Price CS, Forrest GN, Fatkenheuer G, Gareca M, Rehm SJ, Brodt HR, Tice A, Cosgrove SE (2006). Daptomycin versus standard therapy for bacteremia and endocarditis caused by *Staphylococcus aureus*. N Engl J Med.

[19] Moise PA, Smyth DS, El-Fawal N, Robinson DA, Holden PN, Forrest A, Sakoulas G (2008). Microbiological effects of prior vancomycin use in patients with methicillin-resistant *Staphylococcus aureus* bacteraemia. J Antimicrob Chemother.

[20] Rose WE, Rybak MJ, Kaatz GW (2007). Evaluation of daptomycin treatment of *Staphylococcus aureus* bacterial endocarditis: an in vitro and in vivo simulation using historical and current dosing strategies. J Antimicrob Chemother.

[21] Mangili A, Bica I, Snydman DR, Hamer DH (2005). Daptomycin-resistant, methicillin-resistant *Staphylococcus aureus* bacteremia. Clin Infect Dis.

[22] Cui L, Tominaga E, Neoh HM, Hiramatsu K (2006). Correlation between Reduced Daptomycin Susceptibility and Vancomycin Resistance in Vancomycin-Intermediate *Staphylococcus aureus*. Antimicrob Agents Chemother.

[23] Kelley PG, Gao W, Ward PB, Howden BP (2011). Daptomycin non-susceptibility in vancomycin-intermediate *Staphylococcus aureus* (VISA) and heterogeneous-VISA (hVISA): implications for therapy after vancomycin treatment failure. J Antimicrob Chemother.

[24] Saginur R, Stdenis M, Ferris W, Aaron SD, Chan F, Lee C, Ramotar K (2006). Multiple combination bactericidal testing of staphylococcal biofilms from implant-associated infections. Antimicrob Agents Chemother.

[25] Matsuo M, Hishinuma T, Katayama Y, Cui L, Kapi M, Hiramatsu K (2011). Mutation of RNA polymerase beta subunit (*rpoB*) promotes hVISA-to-VISA phenotypic conversion of strain Mu3. Antimicrob Agents Chemother.

[26] Aubry-Damon H, Soussy CJ, Courvalin P (1998). Characterization of mutations in the *rpoB* gene that confer rifampin resistance in *Staphylococcus aureus*. Antimicrob Agents Chemother.

[27] Cui L, Isii T, Fukuda M, Ochiai T, Neoh HM, Camargo IL, Watanabe Y, Shoji M, Hishinuma T, Hiramatsu K (2010). An *RpoB* mutation confers dual heteroresistance to daptomycin and vancomycin in *Staphylococcus aureus*. Antimicrob Agents Chemother.

[28] Benvenuto M, Benziger DP, Yankelev S, Vigliani G (2006). Pharmacokinetics and tolerability of daptomycin at doses up to 12 milligrams per kilogram of body weight once daily in healthy volunteers. Antimicrob Agents Chemother.

[29] Rose WE, Leonard SN, Rybak MJ (2008). Evaluation of daptomycin pharmacodynamics and resistance at various dosage regimens against *Staphylococcus aureus* isolates with reduced susceptibilities to daptomycin in an in vitro pharmacodynamic model with simulated endocardial vegetations. Antimicrob Agents Chemother.

[30] Murray KP, Zhao JJ, Davis SL, Kullar R, Kaye KS, Lephart P, Rybak MJ (2013). Early use of daptomycin versus vancomycin for methicillin-resistant *Staphylococcus aureus* bacteremia with vancomycin minimum inhibitory concentration >1 mg/L: a matched cohort study. Clin Infect Dis.

[CR31] The pre-publication history for this paper can be accessed here:http://www.biomedcentral.com/1471-2334/14/459/prepub

