# Supplementary figures and images for: First case report of vancomycin-intermediate sequence type 72 Staphylococcus aureuswith nonsusceptibility to daptomycin
Source: BMC Infect Dis. 2014 Aug 23;14:459. doi: 10.1186/1471-2334-14-459 (PMC4150982; doi:10.1186/1471-2334-14-459)

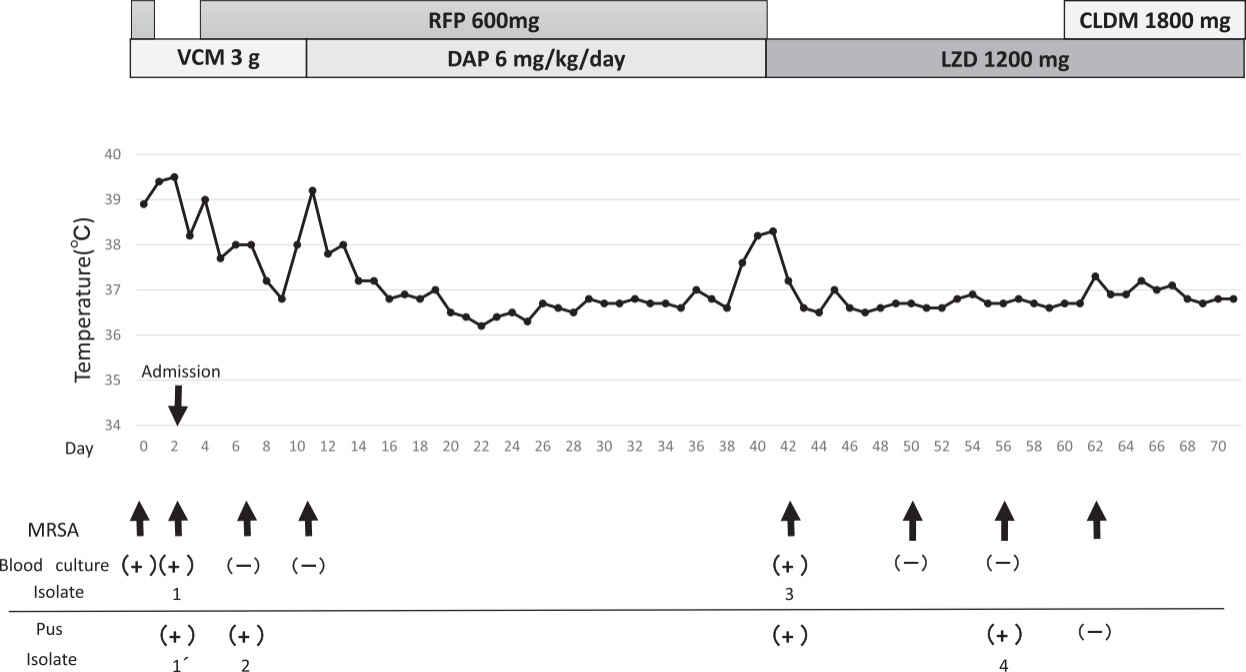

Supplement: Supplementary file 1 — Authors’ original file for figure 1 [file 12879_2014_3751_MOESM1_ESM.pdf]

1      1'      2      3      4      Ladder

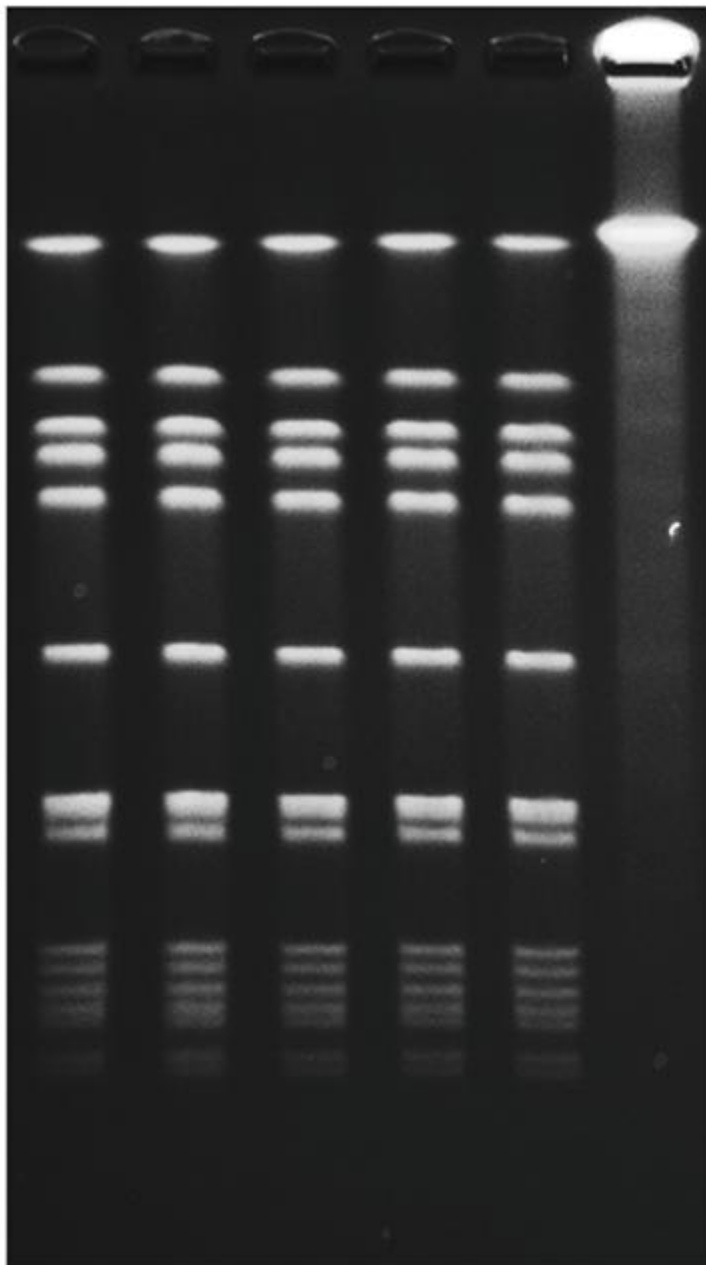

Supplement: Supplementary file 2 — Authors’ original file for figure 2 [file 12879_2014_3751_MOESM2_ESM.pdf]
